# Supplementary material for: Effector Sntf2 Interacted with Chloroplast-Related Protein Mdycf39 Promoting the Colonization of Colletotrichum gloeosporioides in Apple Leaf
Source: Int J Mol Sci. 2022 Jun 7;23(12):6379. doi: 10.3390/ijms23126379 (PMC9224526; doi:10.3390/ijms23126379)
Supplement: Supplementary file 1 [file ijms-23-06379-s001.zip › ijms-1709507-supplementary.pdf]

SUPPLEMENTARY MATERIALS

Additional information can be accessed in the Supporting Information online.

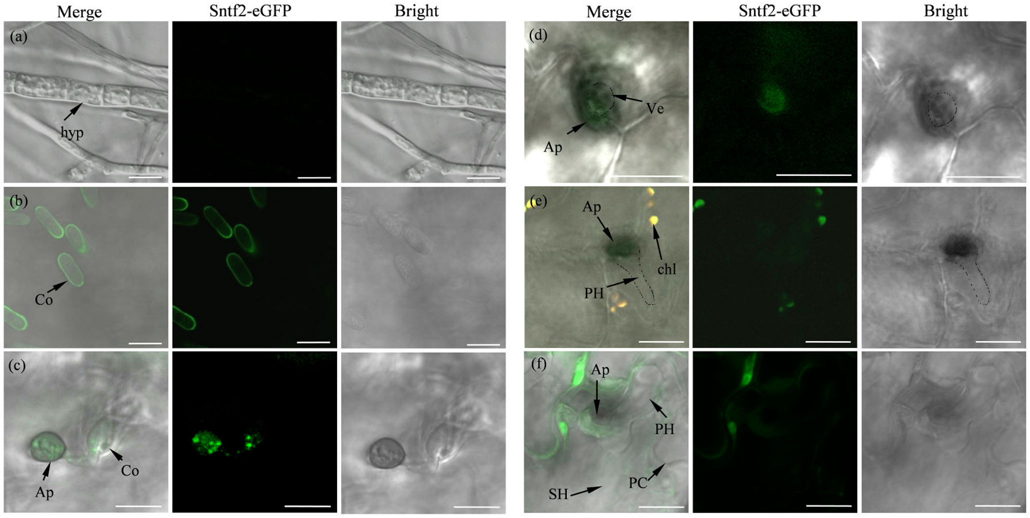

Figure S1. The different infection phases in apple leaves of the Δ*sntf2*-1/*SNTF2p:SNTF2:eGFP* strain. (a) Hyphae phase: hyphae were harvested from 3-day-old PDA cultures. (b) Conidia phase. The picture showed the conidia inoculated on apple leaves. (c) Appressoria formed at 12 h post-inoculation (hpi). (d) Infection vesicles formed at 24 hpi. (e) Primary hyphae formed at 48 hpi. (f) Secondary hyphae formed at 72 hpi. During the infection, the fluorescence signals of Sntf2-eGFP fusion protein were shown in figure b-f. Images were acquired using a confocal microscope. Hyp: hyphae; Co: conidia; Ap: appressorium; Ve: infection vesicle; PH: primary hyphae; SH: secondary hyphae; chl: chloroplast; PC: plant cell. Bar: 10 μm.

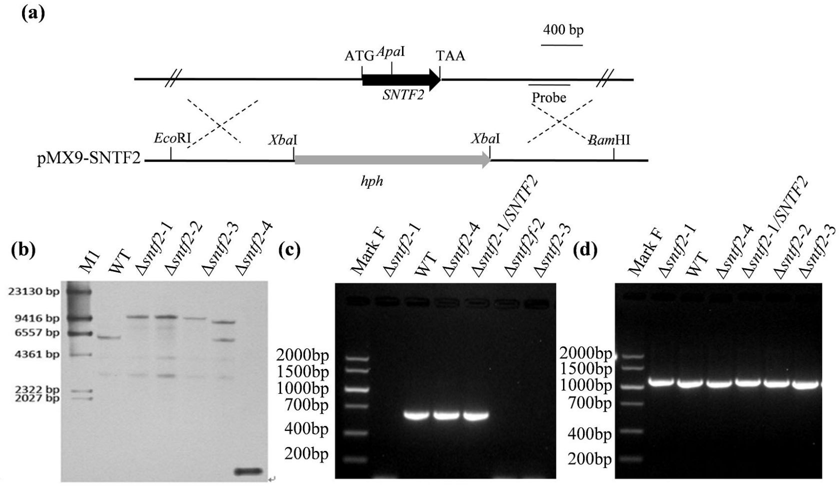

Figure S2. Construction and identification of Δ*sntf2* deletion mutants and Δ*sntf2*-1/*SNTF2* complementation strain. (a) Strategy for deletion of *SNTF2* via homologous recombination. bp, base pairs; *hph*, hygromycin phosphotransferase gene. The restriction enzymes were *Eco*RI, *Xba*I, and *Bam*HI. (b) Southern hybridization confirmed the deletion of *SNTF2*. Total genomic DNA of the wild-type (WT) and Δ*sntf2* mutants (Δ*sntf2*-1, Δ*sntf2*-2, Δ*sntf2*-3, and Δ*sntf2*-4) was subjected *Apa*I digestion and used for Southern blot analysis. Probe, a 400 bp DNA fragment amplified from the genomic DNA of the WT. M1, λDNA/*Apa*I digestion (23130, 9416, 6557, 4361, 2322, and 2027 bp) was used as a molecular weight marker. (c) Transcript detection of the *SNTF2* in the WT, Δ*sntf2*-1, Δ*sntf2*-2, Δ*sntf2*-3, and Δ*sntf2*-1/*SNTF2* strains through PCR analysis. (d) The *β-tubulin* gene was used as the control gene.

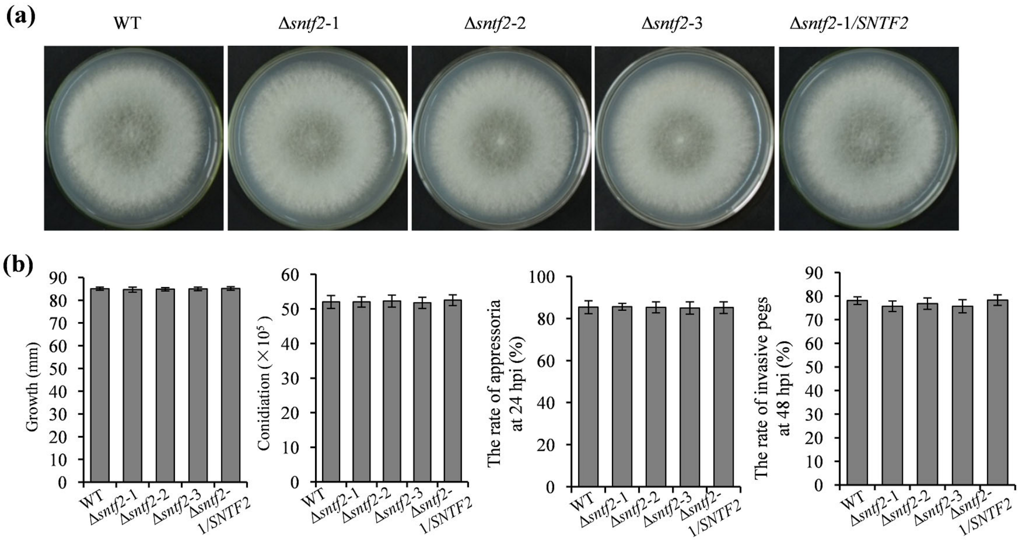

Figure S3. The effects of *SNTF2* deletion on the colony morphology, growth rate, conidial production, appressorial formation, and invasive pegs formation. (a) Colony morphology and growth of the WT, Δ*sntf2*-1, Δ*sntf2*-2, Δ*sntf2*-3, and Δ*sntf2*-1/*SNTF2* strains on PDA plates after 7 days of cultivation. (b) The colony growth, conidial production, and formation rates of appressoria and invasive pegs of the WT, Δ*sntf2*-1, Δ*sntf2*-2, Δ*sntf2*-3, and Δ*sntf2*-1/*SNTF2* strains. Conidia were harvested from PDA plates at cultured 10 days. During apple leaf infection, the formation rates of appressoria and invasive pegs were observed and calculated at 24 hpi and 48 hpi, respectively. Experiments were performed three times. Error bars represent standard deviations.

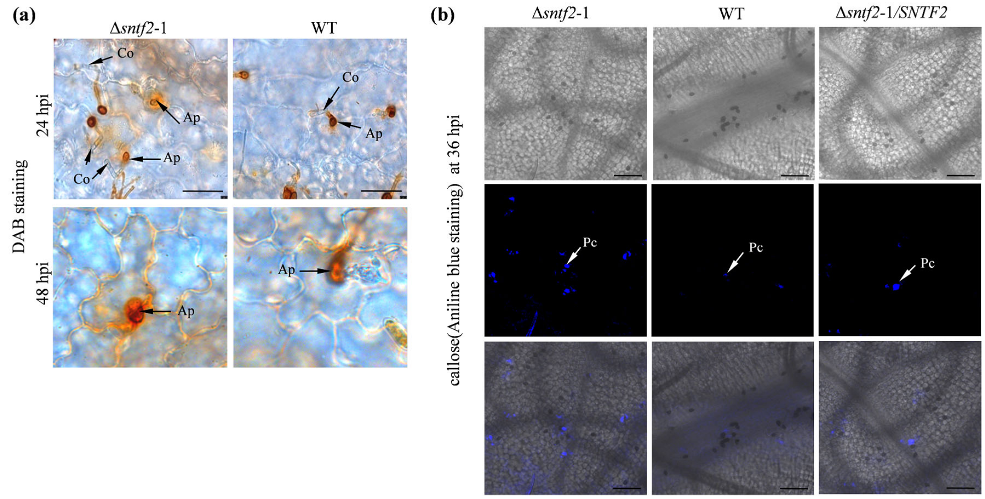

Figure S4. The H_2O_2 accumulation and callose deposition in apple leaves during the infection. (a) DAB staining was performed to detect H_2O_2 accumulation on apple leaves at 24 hpi and 48 hpi. DAB oxidation led to brownish polymer formation, that was deposited at the site of H_2O_2 accumulation. (b) Aniline blue staining was to observe callose deposition on apple leaves at 36 hpi. Apple leaves were inoculated with WT, Δ*sntf2*-1, or Δ*sntf2*-1/*SNTF2* strains. Co: conidia; Ap: appressorium; Pc: callose deposition. Bar: 50 μm.

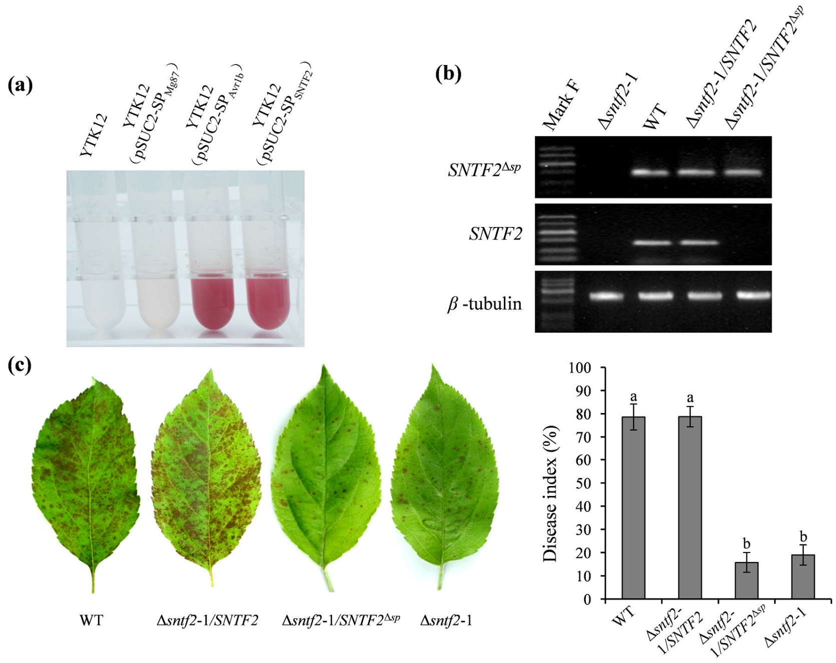

Figure S5. Functional confirmation of the signal peptide of Sntf2. (a) Evaluation of the invertase activity of the transformed YTK12 strains by the reduction of 2, 3, 5-triphenyltetrazolium chloride to insoluble red formazan. (b) Transcripts detection of the *SNTF2* and *SNTF2*^{Δsp} in the WT, Δ*sntf2*-1, Δ*sntf2*-1/*SNTF2*, and Δ*sntf2*-1/*SNTF2*^{Δsp} strains using PCR analysis. The *β*-tubulin gene was used as a control gene. Mark F (2000, 1500, 1000, 700, 400, and 200 bp) was used as a molecular weight marker. (c) Pathogenicity test of the signal peptide deletion mutant. Apple leaves were inoculated with the WT, Δ*sntf2*-1, Δ*sntf2*-1/*SNTF2p:SNTF2*^{Δsp}, and Δ*sntf2*-1/*SNTF2p: SNTF2* strains and imaged after 72 hpi. Pathogenicity was evaluated based on the disease index. Error bars represent standard deviations. Lower-case letters indicate significant differences ($P < 0.01$).

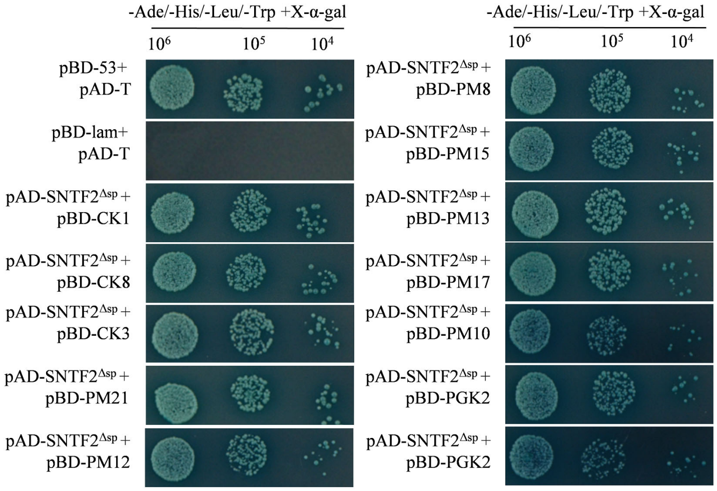

Figure S6. Identification of the interaction proteins of Sntf2 using the yeast two-hybrid assay. Yeast cells Y2Hgold strain transformed with the recombinant vectors were assayed for growth on SD medium deficient in Ade, His, Leu, and Trp with added X-*α*-gal and assayed for LacZ activities on the medium. The Y2Hgold strain harboring pBD-53 (pGBKT7-53) and pAD-T (pGADT7-T) was used as a positive control, and the strain harboring pBD-lam (pGBKT7-Lam) and pAD-T (pGADT7-T) was used as a negative control.

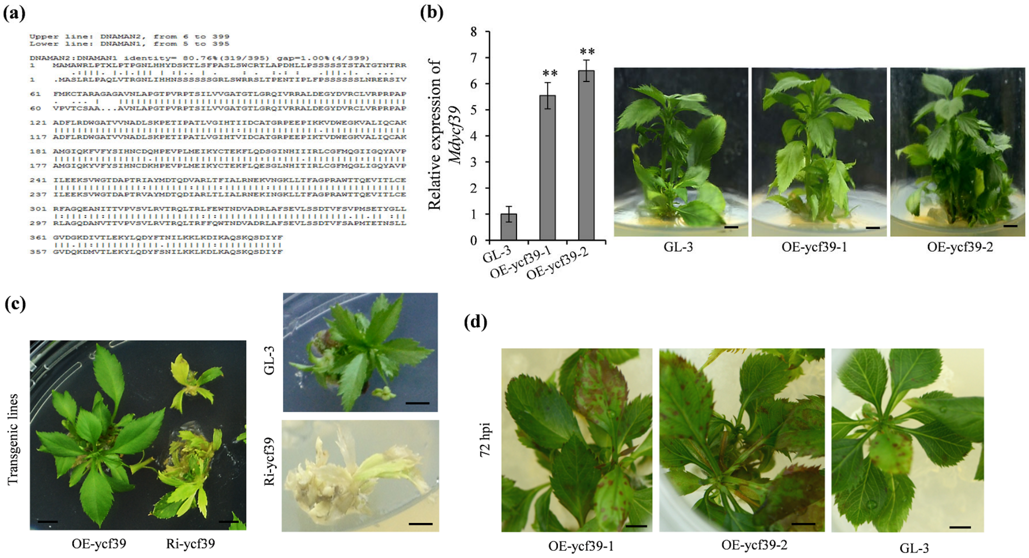

Figure S7. The transgenic plants of *Mdycf39* overexpression and RNA-interference. (a) Alignment of the protein sequences of Mdycf39 and HCF244 using DNAMAN software (version 6). DNAMAN1 represents HCF244; DNAMAN2 represents Mdycf39. (b) The *Mdycf39* overexpression lines and GL-3 were cultured on Murashige and Skoog (MS) medium. Relative expression levels of *Mdycf39* in transgenic lines using qRT-PCR. The vertical axis represents the relative fold-changes of transcripts, compared with the GL-3 sample. Apple leaf *MdUBQ* was used as the reference gene. Error bars represent standard deviations. The double asterisks indicate statistically significant differences (one-way analysis of variance, $P < 0.01$). (c) *Mdycf39* overexpression transgenic lines, RNA-interference tissues, and control plant GL-3 were cultured on MS medium. The *Mdycf39* silencing lines was not grow normally on MS medium with pale white leaves. (d) The susceptibility analysis of *Mdycf39* overexpression lines to *Colletotrichum gloeosporioides*. Bar: 5 mm.

Table S1. The Sntf2-interacting proteins screened from 'Golden Delicious' by Yeast two-hybrid assays.

Name	Gene ID*	Annotation	Times
Ck1 (Mdycf39)	MD05G1131800	uncharacterized protein ycf39-like	10
CK8	MD06G1140000	gamma carbonic anhydrase-like 2, mitochondrial	5
CK3	MD15G1137600	uncharacterized protein	3
PM12	MD05G1115700	triosephosphate isomerase, chloroplastic	5
PM21	MD15G1269900	probable galacturonosly transferase-like 1	5
PM8	MD15G1411600	chlorophyll a-b binding protein CP24 10A, chloroplastic	5
PM15	MD03G1069500	beta-glucosidase 13-like	2
PGK2	MD10G1265400	chlorophyll a-b binding protein of LHCII type 1	5
PGK1	MD13G1247100	uncharacterized protein	2
PM13	MD06G1122300	uncharacterized protein	2
PM17	MD03G1086300	ribulose bisphosphate carboxylase/oxygenase activase, chloroplastic isoform X2	3
PM10	MD06G1055800	3-isopropylmalate dehydratase small subunit 3-like	2

* The genes were from *Malus x domestica* GDDH13 v1.1 ('Golden Delicious' doubled-haploid tree) genome.

Table S2. Primers used in this study.

Primer	Sequence（5′→3′）	Purpose
**215N1**	AAAGTCGACATGCGTCCATCAACCCT	For constructing the pGR106-S*NTF2* vector
**215N2**	AAAGTCGACTTAAGACATTTGCAGAG-	

	TTTCCT	
**215D1**	AAAGAATTCGCCTCCATAACATGCACA	Amplifying the upstream sequence of the *SNTF2* for constructing the *SNTF2* knock-out vector
**215D2**	AAATCTAGAGTCCCTTGCAGCTATTAC	
**215D3**	AAATCTAGACTATCCAGAGCAACGATGT	Amplifying the downstream sequence of the *SNTF2* for constructing the *SNTF2* knock-out vector
**215D4**	AAAGGATCCGTCCTGTCTCATCATCCCA	
**BOX3**	AAATCTAGAAGAAGATGA-TATTGAAGGAG	Amplifying *hph* for constructing the *SNTF2* knock-out vector
**BOX4**	AAATCTAGAAAGAAGGATTAC-CTCTAAAC	
**HYG-F**	ATGAAAAAGCCTGAACTCAC	validation of *hph* gene in deletion mutants
**HYG-R**	CTATTCCTTTGCCCTCG	
**215F3**	AAAGGATCCACAATGACAACAACAACA AACT	validation of *SNTF2* deletion in mutants
**215R3**	AAATCTAGAGACATTTGCAGAG-TTTCCTAAC	
**TubF**	CTTCCGGCAACAAGTACGT	PCR for β-tubulin
**TubR**	GCGTCCTGGTATTGCTGGT	
**215FC**	AAAGTCGACAAAGATGATTTGATAGTAC	Amplifying the putative promoter and the *SNTF2* sequence for constructing complementation vector
**215R3**	AAATCTAGAGACATTTGCAGAG-TTTCCTAAC	
**215P1**	CCTGGGGTCCATTCGG	Synthesis Southern blot probe
**215P2**	TACGCGGAGTGGGCGAA	
**215G1F**	ATACAATTCTACGGTGCCGGCA	qRT-PCR for *SNTF2*
**215G1R**	AGAAATCAGACGCGTCTTCAGGTC	
**215G2F**	ACACCCCAATCACCTCCACCAA	qRT-PCR for *SNTF2*
**215G2R**	AAGGTGTCGTCGTAGCTCGT	
**215F6**	AAAGAATTCATGCGTCCATCAACCCT	Amplifying the signal peptide (*SP*) sequence of *SNTF2* for constructing pSUC2-SP$_{SNTF2}$ vector
**215R6C**	AAACTCGAGTGTCCTGCCGTGCGCT	
**P215F**	AAACTCGAGCATCTTCCTGGGTGGTCAA	Amplifying the putative promoter sequence of *SNTF2* for constructing *SP* deletion vector
**P215R**	AAATCTAGATTT-GGATGAGGTAGATGTTGAGA	
**215m**	AAATCTA-GAATGACAATGACAACAACAACAAACT	Amplifying the sequence of *SNTF2* without *SP* for constructing SP deletion vector
**215R3**	AAATCTAGAGACATTTGCAGAG-TTTCCTAAC	
**215F4**	TTTAAGCTTATGCGTCCATCAACCCT	validation of *SNTF2 SP* sequence deletion in mutants
**215R3**	AAATCTAGAGACATTTGCAGAG-TTTCCTAAC	
**215BD**	AAA-GAATTCATGACAATGACAACAACAACA AACT	Amplifying the coding sequence of Sntf2 (without *SP*) for constructing pGBKT7-SNTF2$^{\Delta SP}$ vector
**F215Y2**	AAAGTCGACTTAGACATTTGCAGAG-TTTCCTAAC	
**215BD**	AAA-GAATTCATGACAATGACAACAACAACA AACT	Cloning the coding region of Sntf2 (without *SP*) to pGADT7-AD for constructing pAD-SNTF2$^{\Delta SP}$ vector
**215RN**	AAACTCGAGGACATTTGCAGAG-TTTCCTAAC	

Continued to Table S2

Primer	Sequence（5′→3′）	Purpose
**215F3**	AAAGGATCCACAATGACAACAACAACAAACT	Cloning the coding sequence of Sntf2 (without *SP*) to pGR35s-eGFP and pGR35s-YFP$_{1-173}$ vectors
**215R3**	AAATCTAGAGACATTTGCAGAG-TTTCCTAAC	
**215F3**	AAAGGATCCACAATGACAACAACAACAAACT	Cloning the coding sequence of Sntf2 (without *SP*) to pQE30-eGFP for expression Sntf2$^{\Delta SP}$: eGFP:His protein
**F215Y2**	AAAGTCGACTTAGACATTTGCAGAG-TTTCCTAAC	
**C1FM**	AAAGAGCTCATGGCAATGGCTTGGAGG	Cloning the coding sequence of Mdycf39 to pQE30-Flag for expression Mdycf39: Flag protein
**PC1R**	AAAGTCGACTCAGAAGTAAA-TATCAGATTGC	
**215CK1F**	AAAGAATTCATGGCAATGGCTTGGAG	Amplifying the coding sequence of *Mdycf39* for constructing pBD-Mdycf39 vector
**215CK1R**	AAAGTCGACAAAAACAAAATGGTTT-GATTGGGC	
**215C1F**	AAACTCGAGATGGCAATGGCTTGGAGG	Cloning the coding sequence of *Mdycf39* to pGR35s-TagRFP, pGR35s-eGFP, and pGR35s-YFP$^{173-238}$ vectors
**215CK1RM**	AAATCTAGAAAAAACAAAATGGTTT-GATTGGGC	
**215C1F**	AAACTCGAGATGGCAATGGCTTGGAGG	Cloning the *Mdycf39* to pRPHA vector
**215CK1RM**	AAATCTAGAAAAAACAAAATGGTTT-GATTGGGC	
**PC1FN**	AAAGTCGACAAGGTAGATTGGGAR-GGAAAAG	Cloning the partial sequence of *Mdycf39* to pRNAi vector
**PC1RM**	AAATCTAGATACATCCTGGGTGTCCATGT	
**PC2FM**	TTTCTGCAGTACATCCTGGGTGTCCATGT	
**PC2RM**	TTTAGATCTAAGGTAGATTGGGAR-GGAAAAG	
**CK1F**	AAAGTCGACGTCAATGGGAA-GCTTCTCAC	Cloning the partial sequence of *Mdycf39* to pRNAi vector
**215C1RM**	AAATCTAGAGAAGTAAA-TATCAGATTGCTTTG	
**CK1FM**	TTTCTGCAGGAAGTAAA-TATCAGATTGCTTTG	
**CK1R**	TTTAGATCTGTCAATGGGAAGCTTCTCAC	
**PM3F**	TTTGAATTCATGTATCACGCCAA-GAAGTTC	Amplifying the sequence of *PM13* for constructing pBD-PM13 vector
**PM3R**	TTTGTCGACTTATTGTTTTGAAC-CACACAAGGC	
**215PP10F**	TTTGAATTCATGGCCGGCGCC	Amplifying the sequence of *PM10* for constructing pBD-PM10 vector
**215PP10R**	TTTGTCGACTTAACAAGTCAAAGTAG-GAATCATCCC	
**ppm8F**	TTTGAATTCATGGCGGCAACCACC	Amplifying the sequence of *PM8* for constructing pBD-PM8 vector
**ppm8R**	TTTGTCGACTTACAAGCCAAGAGCAC-CAAG	
**ppm12F**	TTTGAATTCATGGCGGTGGCCTCC	Amplifying the sequence of *PM12* for constructing pBD-PM12 vector
**ppm12R**	TTTGTCGACTTAAGCAGCAACTTTCTT-GGC	
**215PP15F**	TTT-GGATCCAAATGTTCAGACTTAACTCGGTG	Amplifying the sequence of *PM15* for constructing pBD-PM15 vector
**215PP15R**	TTTGTCGACTTATCCAAGGAAATATTT-GAACCAGC	
**PP21F**	TTT-GAATTCATGCTCTCTTTCTTCATCAGCT	Amplifying the sequence of *PM21* for constructing pBD-PM21 vector
**PP21R**	TTTAAGCTTTCAAGACTCCAGCGCGAAG	

**215CP8F**	TTTCATATGATGGCAGCAATAGCTCG	Amplifying the sequence of *CK8* for constructing pBD-CK8 vector
**215CP8RM**	TTT-GGATCCTTAAATGGAAATCCCCAAA-GACT	
**215CP3F**	TTTGAATTCATGCTGACCCTTCACTTTC	Amplifying the sequence of *CK3* for constructing pBD-CK3 vector
**215CP3RM**	TTTGTCGACTTACTGCAAAGCATT-AGAAACAG	

Continued to Table S2

Primer	Sequence（5′→3′）	Purpose
**215PPGF**	TTTGAATTCATGGCTGCTTCCACAATG	Amplifying the sequence of *PGK2* for constructing pBD-PGK2 vector
**215PPGR**	TTTGTCGACTTACTTTCCGGGAACGAAGTTT	
PPGKF	TTTGAATTCATGTCGTCCTCCATATCCTC	Amplifying the sequence of *PGK1* for constructing pBD-PGK1 vector
PPGKR	TTTGTCGACTTAG-TAATCATCTTCTTCTTCGTCTAT	
**215pm1FM**	TTTCATATGATGGCCGCCATGCACAC	Amplifying the sequence of *PM17* for constructing pBD-PM17 vector
**215pm1RM**	TTTGTCGACTTAACTTGTTGGGCCGCCTTTC	
**MdUBQ-F**	CTCCGTGGTGGTTTTTAAGT	qRT-PCR for *MdUBQ*
**MdUBQ-R**	GGAGGCAGAAACAGTACCAT	
**GC1F**	ATCTTCTTCCACCTCCACCGC	qRT-PCR for *Mdycf39*
**GC1R**	TGTCCCCGGAGCAAGATTCACAG	
**GC3F**	GCAAACCAGAGACCATACCTGC	qRT-PCR for *Mdycf39*
**GC3R**	TTTTATGGGCTCTTCGGGACGG	
